# Non-functional paraganglioma of the urinary bladder: a case report

**DOI:** 10.1186/1752-1947-4-216

**Published:** 2010-07-19

**Authors:** Dan-Feng Xu, Ming Chen, Yu-Shan Liu, Yi Gao, Xin-Gang Cui

**Affiliations:** 1 Department of Urology, Changzheng Hospital, Second Military Medical University, Shanghai 200003, China

## Abstract

**Introduction:**

Paragangliomas that originate from the urinary bladder are extremely rare. In most series, bladder paragangliomas often cause micturitional attacks. Treatment modalities include transurethral resection and cystectomy (partial or total). Prognosis of bladder paraganglioma is similar to that of adrenal pheochromocytoma.

**Case presentation:**

A 55-year-old Chinese woman presenting with the sole complaint of lower abdominal pain for one month was admitted to our hospital. Ultrasound and computed tomography revealed a mass on the dome of the bladder measuring 4.0 × 3.0 cm. The tumor was completed removed by laparoscopic partial cystectomy. Histological examination of the tumor indicated paraganglioma of the urinary bladder. The clinical features, diagnosis, management and pathological findings of paraganglioma of the urinary bladder are discussed.

**Conclusions:**

Bladder paraganglioma should be considered as a differential diagnosis to neoplasm in the urinary bladder, although there is no characteristic symptom. Laparoscopic partial cystectomy may be the first choice in treating paraganglioma of the urinary bladder, offering several advantages such as less invasion, rapid recovery and early discharge from the hospital.

## Introduction

Paragangliomas, which arise from the chromaffin tissue of the sympathetic nervous system in locations outside the adrenal gland, are referred to as extra-adrenal pheochromocytomas. The first case of paraganglioma of the urinary bladder was reported by Zimmerman in 1953 [1]. Paragangliomas of the urinary bladder account for 0.06% of all bladder tumors and 6% of extra-adrenal pheochromocytomas [2]. In most series, paraganglioma of the urinary bladder often causes micturitional attacks, including headache, palpitations, fainting and visual disturbances. Because of its low incidence rate, the prognosis of bladder paraganglioma is not well established. However, some reports suggest that paraganglioma has the same prognosis as adrenal pheochromocytoma. Here we describe a 55-year-old Chinese woman with non-functional paraganglioma of the urinary bladder, found after a submucosal mass was removed by laparoscopic partial cystectomy.

## Case presentation

**Figure 1:**
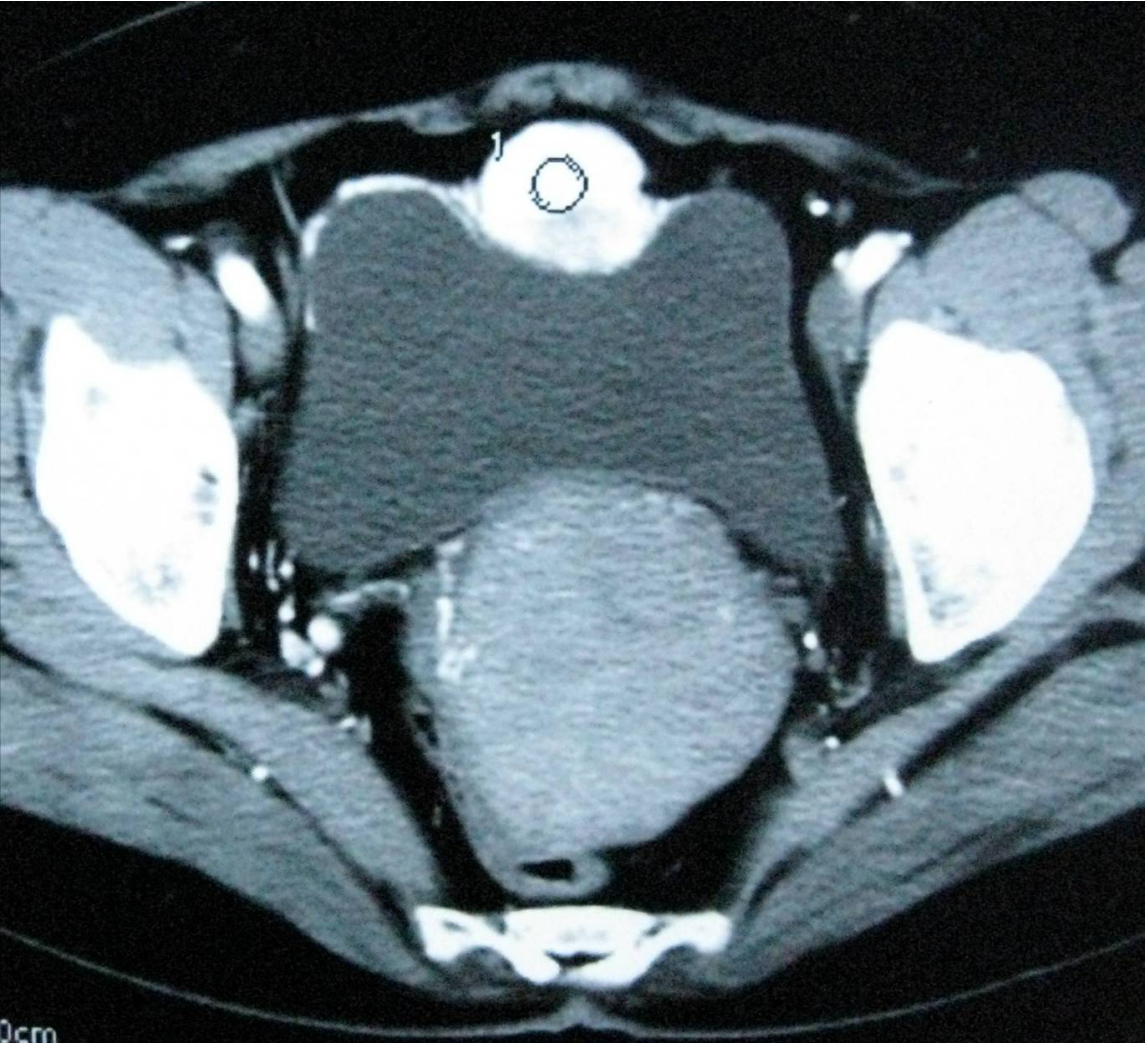
**CT scan showing a nodular mass with intensive enhancement on the dome of bladder**.

A 55-year-old Chinese woman complaining of lower abdominal pain for one month was admitted to our hospital. Her family history was unremarkable and she had no previous medical problems. Her blood pressure was 110-130/70-80 mmHg, and her heart rate was in the normal range. Physical examination showed no evidence of hypertensive disease. Both ultrasound examination and computed tomography (CT) scan demonstrated a mass on the dome of the bladder, measuring 4.0 × 3.0 cm (Figure 1). Our patient underwent cystoscopic examination and a solitary submucosal mass was seen on the dome of the bladder, with normal mucosal covering. No sign of any metastatic disease was found on ultrasound examination or CT scans of other abdominal organ systems. Routine blood and urine tests showed no abnormality. On the basis of the first diagnosis of bladder tumor, our patient was admitted for laparoscopic partial cystectomy. During the intervention, no hypertension or massive bleeding occurred. Post-operative recovery was uneventful. At 10 months follow-up, our patient felt well, her blood pressure was normal without receiving anti-hypertensive medication.

**Figure 2:**
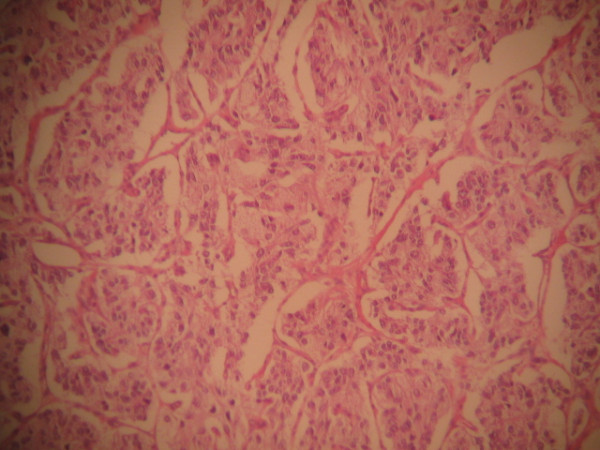
**The tumor cells grow in a nested, zellballen pattern (hematoxylin and eosin ×100)**.

**Figure 3:**
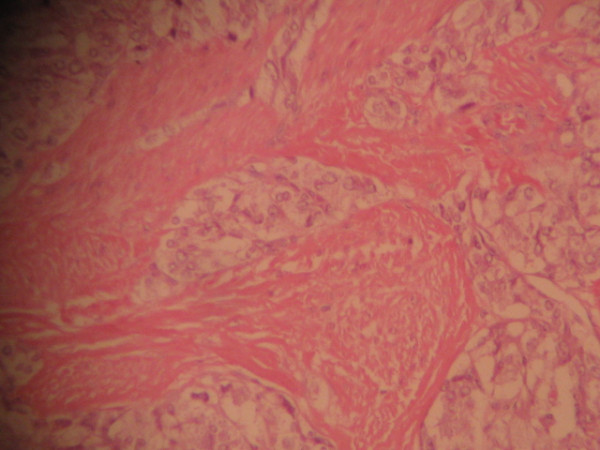
**The tumor cells invade the muscular layer (hematoxylin and eosin ×100)**.

**Figure 4:**
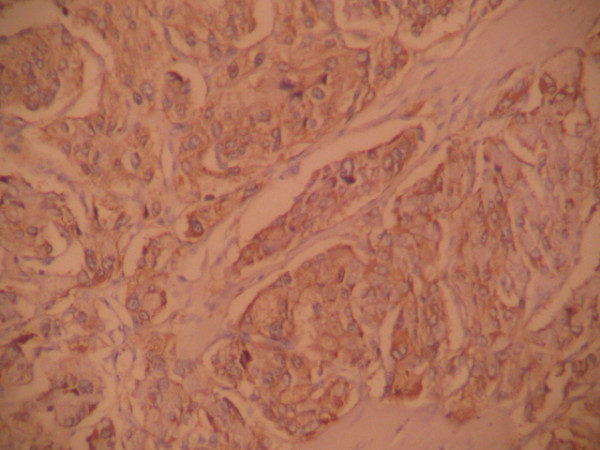
**Immunostaining for synaptophysin is strongly positive (DAB ×100)**.

**Figure 5:**
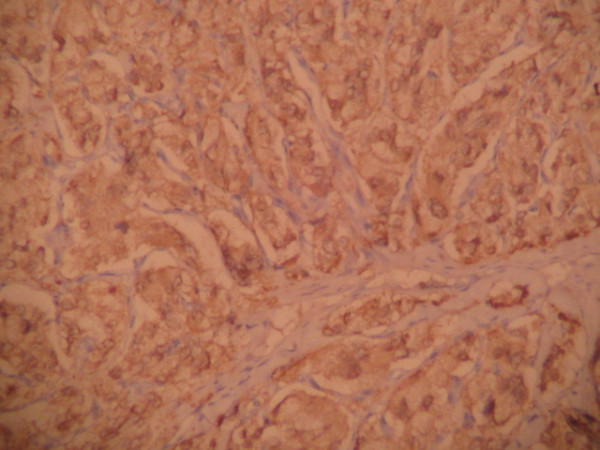
**Immunostaining for NSE is strongly positive (DAB ×100)**.

Pathological evaluation revealed paraganglioma to be the final diagnosis. On histopathologic examination, the tumor cells were arranged in a nested pattern (Figure 2). The tumor had invaded the muscular wall of the bladder (Figure 3). The tumor cells showed strong positive enhancement with synaptophysin (Figure 4) and neuron-specific enolase (NSE) (Figure 5), while immunostaining for chromogranin was negative.

## Discussion

The origin of paraganglioma of the urinary bladder is still unclear. Previous reports show a slight preponderance in females (female/male ratio is 3:1) during the second to fourth decades of life [3]. The case described in our report was a 55-year-old Chinese woman. In former series, patients may present with headache, palpitations and paroxysmal hypertension due to catecholamine excess, especially during micturition. So when the presence of paraganglioma of the urinary bladder is suspected, endocrine tests should be performed, including vanillyl mandelic acid (VMA) in 24 hour urine sample, serum epinephrine and so on. Surprisingly, our patient had none of these symptoms, so we did not carry out any endocrine tests. However, even in those cases with typical symptoms, not all cases have elevated plasma or urine catecholamine or its metabolites if the samples are not collected at the time the micturitional attacks occurred.

With the advances in imaging technology, ultrasound, computed tomography (CT) and magnetic resonance imaging (MRI) may be useful in localizing the tumor, while I^131^-methyliodobenzylguanidine (I^131^-MIBG) and positron emission tomography (PET)-CT help to evaluate its function. The significance of cystoscopy is limited and biopsy is not recommended since the tumor is usually located in the submucosa with an intact surface, and biopsy may provoke a hypertensive episode in patients who have not had proper medical treatment. Additionally, artifactual changes in a small biopsy may confuse diagnosis [4]. In our case, we did not schedule a biopsy, because the CT scan showed intense vessel density, which gave us the misleading impression of hemangioma.

In some asymptomatic bladder paragangliomas, it is very difficult to have an actual pre-operative diagnosis. Histological and immunohistochemical diagnosis become the last choices. Paragangliomas of the urinary bladder show histological features similar to adrenal pheochromocytomas. The tumor cells usually grow in a nested, zellballen pattern with delicate fibrovascular stroma, although they may also grow in a diffuse pattern [5]. Immunohistochemical staining is required for a definitive diagnosis. Chromogranin, synaptophysin and NSE can help to identify neural tissue and neuroendocrine cells [6]. A marked strong reaction with synaptophysin and NSE was seen in our case but the tissue was negative for chromogranin.

Treatment modalities include transurethral resection and partial or total cystectomy combined with pelvic lymph node dissection, especially in the presence of proven metastasis [7]. However, the optimal management mode is still uncertain. For patients who had characteristic paroxysmal hypertension during micturition, it is necessary to stabilize hypertension before the operation by using alpha-blocking agents for about two weeks and expanding the blood volume, which is similar to treatment for other pheochromocytomas. Sometimes it is hard to have a definitive pre-operative diagnosis, resulting in insufficient preparation, which complicates the transurethral resection because unexpected intra-operative hypertensive crisis and bleeding may occur [8]. With the improvements seen in laparoscopy technique, laparoscopic partial cystectomy becomes the treatment of choice. Dilbaz *et al. *[9] first reported a case that was finished by using the laparoscopic approach. In our case, we successfully performed a laparoscopic partial cystectomy. Intra-operative blood pressure was stable during the operation, and the margins were negative for tumor.

## Conclusions

Bladder paraganglioma may be misdiagnosed when characteristic symptoms are absent. Laparoscopic partial cystectomy for bladder paraganglioma may be the first treatment of choice, offering several advantages such as less invasion, rapid recovery and early discharge from the hospital.

## Competing interests

The authors declare that they have no competing interests.

## Authors' contributions

DFX performed the surgery. MC collected the patient data and wrote the manuscript. YSL was a major contributor in writing the manuscript. YG performed the surgery as first assistant. XGC performed the surgery as second assistant. All authors read and approved the final manuscript.

## Consent

Written informed consent was obtained from the patient for publication of this case report and any accompanying images. A copy of the written consent is available for review by the Editor-in-Chief of this journal.
